# GlobeCorr: interactive globe-based visualization for correlation datasets

**DOI:** 10.1093/bioadv/vbac099

**Published:** 2023-01-06

**Authors:** Mariam Arab, Nolan Woods, Emma S Garlock, Geoffrey L Winsor, Jaclyn P Parks, Baofeng Jia, Dany Doiron, Tim K Takaro, Jeffrey R Brook, Fiona S L Brinkman

**Affiliations:** Molecular Biology and Biochemistry, Simon Fraser University, Burnaby, BC V5A 1S6, Canada; Molecular Biology and Biochemistry, Simon Fraser University, Burnaby, BC V5A 1S6, Canada; Molecular Biology and Biochemistry, Simon Fraser University, Burnaby, BC V5A 1S6, Canada; Molecular Biology and Biochemistry, Simon Fraser University, Burnaby, BC V5A 1S6, Canada; Faculty of Health Sciences, Simon Fraser University, Burnaby, BC V5A 1S6, Canada; Cancer Control Research, BC Cancer Research Institute, Vancouver, BC V5Z 1L3, Canada; Molecular Biology and Biochemistry, Simon Fraser University, Burnaby, BC V5A 1S6, Canada; Research Institute of McGill University Health Centre, Montreal, QC H4A 3J1, Canada; Faculty of Health Sciences, Simon Fraser University, Burnaby, BC V5A 1S6, Canada; Dalla Lana School of Public Health, University of Toronto, Toronto, ON M5T 3M7, Canada; Molecular Biology and Biochemistry, Simon Fraser University, Burnaby, BC V5A 1S6, Canada

## Abstract

**Motivation:**

Increasingly complex omics datasets are being generated, along with associated diverse categories of metadata (environmental, clinical, etc.). Looking at the correlation between these variables can be critical to identify potential confounding factors and novel relationships. To date, some correlation globe software has been developed to aid investigations; however, they lack secure, dynamic visualization capability.

**Results:**

GlobeCorr.ca is a web-based application designed to provide user-friendly, interactive visualization and analysis of correlation datasets. Users load tabular data listing pairwise variables and their correlation values, and GlobeCorr creates a dynamic visualization using ribbons to represent positive and negative correlations, optionally grouped by domain/category (such as microbiome taxa against other metadata). GlobeCorr runs securely (locally on a user’s computer) and provides a simple method for users to visualize and summarize complex datasets. This tool is applicable to a wide range of disciplines and domains of interest, including the bioinformatics/microbiome and metadata examples provided within.

**Availability and Implementation:**

See https://GlobeCorr.ca; Code provided under an open source MIT license: https://github.com/brinkmanlab/globecorr.

## 1 Introduction

Multiple correlation analysis and confounder identification are increasingly used in bioinformatics, as microbiome and other omics data are examined in the context of other complex data (sometimes referred to as ‘metadata’). However, many of these methods rely on a priori knowledge of possible confounders, and one cannot rely solely on statistical tests (Lee and Burstyn, 2016; VanderWeele and Shpitser, 2011). Researchers use static heat maps as a cursory tool to visualize and identify correlated variables in a dataset; however, the colour scales can make differentiating significant from non-significant correlations challenging. Large matrices can also become laborious and time-consuming to analyse. For genomics, valuable Circos plots have been established to effectively depict relationships in rich datasets (Krzywinski *et al.*, 2009).

To complement Circos plots, Patel and Manrai (2014) introduced the concept of exposome globes for mapping environment-wide associations. Exposome globes, or more generally correlation globes, can aid the identification of confounders and provide a relatively simple way to visualize very complex datasets. More recently, Chung *et al.* (2018) updated and released the R library for generating these exposome globes using Circlize. However, these plots that are generated are confined to a static figure and not suitable for users who do not have experience in R programming. Nevertheless, with the introduction of R Shiny apps (Chang *et al.*, 2022), some visualization R packages, have been adapted to provide correlation matrices through the web page. However, no packages currently allow the interactive visualization of exposome globes through R Shiny. Furthermore, R Shiny apps require the user to upload data to a server, which may violate privacy restraints in some cases. Other tools that can be used to plot correlation networks (e.g. cytoscape; Ono, 2022), are not intuitive for novice users and include other functions not relevant to creating correlation globes, further adding to its usage complexity. In many areas of research, for example, large-scale studies combining metagenomics, environmental and/or clinical datasets, there is a need for correlation visualization tools that are user-friendly, secure and dynamic to help researchers explore their complex data.

## 2 Overview

GlobeCorr is a simple, efficient, user-friendly web platform, accessible through any browser, to create interactive and dynamic correlation globes that requires no user knowledge of programming. It is written using the Vue framework (https://vuejs.org), while the visualizations are made using the amCharts4 javascript library (https://amcharts.com). GlobeCorr exists only as visualization software and does not perform any statistical analyses on the provided data. GlobeCorr plots are generated locally, with no data uploaded or stored on its servers. This allows the added capability of being run securely offline. The use of interactive visualizations enables a more extensive investigation of correlations and facilitates the identification of new hypotheses generated from analyses of large datasets that are difficult to visualize using a static figure.

## 3 Features

### 3.1 Data import

GlobeCorr uses the PapaParse.js library (http://papaparse.com) to read comma-delimited files with columns containing the information (in order) variable1, variable1 domain, variable2, variable2 domain and correlation coefficient. For proper rendering, the file must have a header line of exactly ‘variable1, var1_domain, variable2, var2_domain, coef’.

### 3.2 Visual features and layout

By default, domains around the edge of the globe are arranged by size. Correlation bands between variables stretch across the globe and are coloured according to positive or negative correlation value. Hovering your mouse over a band will display the associated variables and their coefficients and moving your mouse over a domain will highlight all correlations with that domain. There are several options available for users to customize and interact with the layout and aesthetics of their globe: one may plot domains based on domain size or the input order found in their spreadsheet/CSV file, or change the orientation of the domains by selecting the domain of interest and rearranging it around the circumference of the circle. A user may also exclude a domain by clicking on it with the arc, or only visualize more significant correlations above a specified absolute value (i.e. only correlations that are over 0.3 or under −0.3).

### 3.3 Accessibility and aesthetics

Text and interactive elements can be made accessible using the wide range of colour customization tools available to ensure that viewers who cannot see the full-colour spectrum can use GlobeCorr. Additionally, items in focus display rollover tooltips, and hover events are registered. This is helpful for users who depend on alerts and announcements for dynamic on-screen events. Re-aesthetics, one may adjust the font size or label width for the domain labels, plus positive and negative correlation bands can have their properties changed independently. Band colour and background can be adjusted to match a preferred colour palette for export. A user can also download static versions of the visualized data in PNG, SVG, JPG or PDF format. The visualization settings can be easily retained via copy and paste of a text field that maintains JSON encoded values of the settings.

## 4 Example analyses

**Fig. 1.**
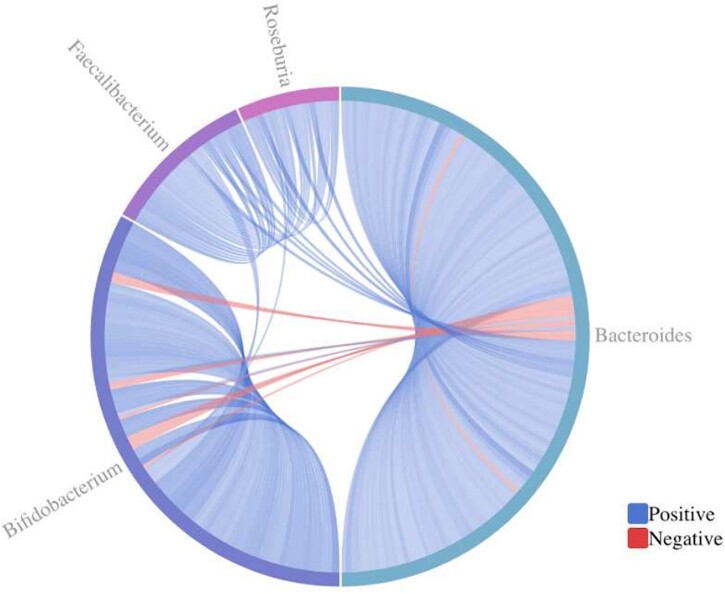
Sample GlobeCorr plot for a co-occurrence network of gut bacteria from (Das *et al.*, 2018). The correlation threshold has been set to 0.4 (±0.4). To explore specific correlations, users may hover over bands of interest to see relevant variable information. Users see that the majority of correlations are intra-domain and positive, with all negative correlations associated with the genus Bacteroides. See text (Section 4) to view the dynamic visualizations using GlobeCorr

**Fig. 2.**
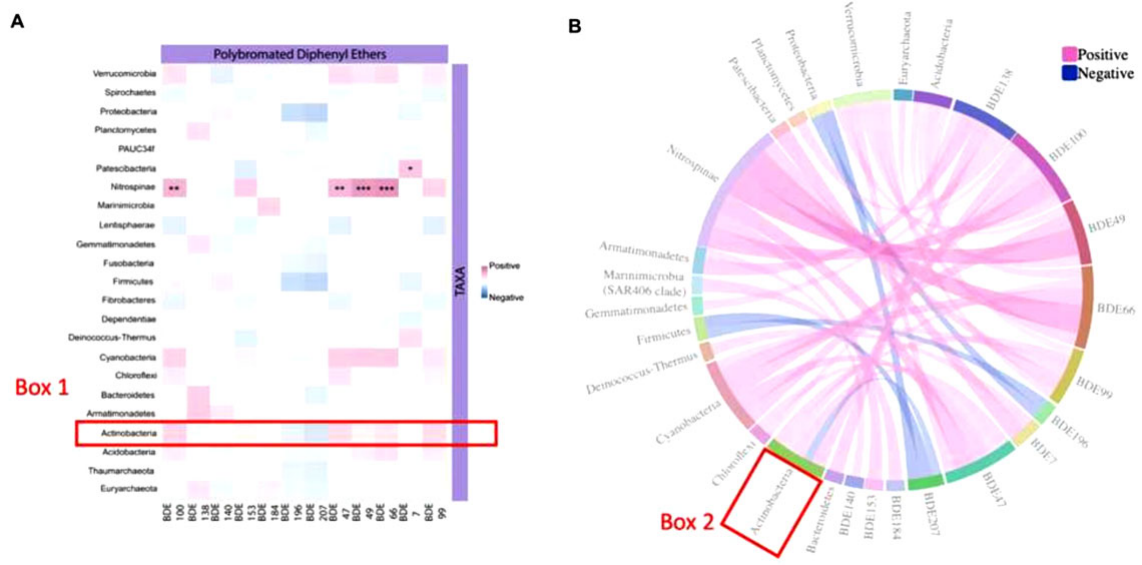
(**A**) Heat map depicting the relationship between Beluga (*Delphinapterus leucas*) skin microbiome (shown as domain; phylum) and beluga blubber contaminants. Blubber contaminants are arranged along the horizontal, while microbiome comments are shown along the vertical at the phylum level. A diverging colour scale is used to show the strength of the positive and negative correlations in different colours. No cutoff is applied to the correlation coefficients, as is typical for such analyses. The significance for correlation is indicated by: ***P < 0.001, **P < 0.01, *P < 0.05. (**B**) Sample GlobeCorr plot depicting the relationship between Beluga skin microbiome and blubber contaminants with a correlation cutoff of |0.2|. Blubber contaminants are located on the right and side of the image and microbiome components are depicted on the left at the phylum level. See text (Section 4) to view the dynamic visualizations using GlobeCorr

Two example analyses are provided and shown in Figures 1 and 2 and can be viewed dynamically at https://globecorr.ca/globe?view=/supplemental_globe_0603.csv&threshold=0.2&sort=none and https://globecorr.ca/globe?view=/taxa_long0514.csv&threshold=0.4.

Figure 1 shows an example of GlobeCorr analysis of a co-occurrence network analysis of gut bacterial species in humans, using a correlation threshold cutoff of 0.4. This rich dataset comprises over 600 correlations to assess (Das *et al.*, 2018). Using GlobeCorr, one can draw some basic conclusions and complement the figures from the (Das *et al.*, 2018) paper. For example, one can observe that the majority of the correlations are intra-domain and positive, but that all negative correlations are associated with the genus Bacteroides. An additional example, exploring the correlations between the beluga skin microbiome and blubber contaminants, illustrates the benefit of a globe-based analysis, including how it complements a heat map. One can view more interactive examples, plus the tutorial on GlobeCorr.ca.

Another example of a microbiome/environmental contaminant correlation analysis is provided in Figure 2. Here, correlation data for beluga skin microbiome components and polybrominated diphenyl ethers in beluga blubber are shown. This dataset (Jia *et al.*, 2022) illustrates the utility of heat maps and GlobeCorr diagrams on the same dataset. Traditional heat maps and GlobeCorr diagrams complement each other. Using heat maps, a user can more easily see the range of correlations present in the dataset if they are using a continuous colour scale. It is also easier to add visual indicators of significance, as shown in Figure 2A, with the asterisks. However, trends in individual domains are easier to evaluate and summarize from the Globecorr visualization (Fig. 2B) as they are all shown close together instead of having to trace a long column up the heatmap (see example comparison shown in Fig. 2 Boxes 1 and 2 for the phylum Actinobacteria). GlobeCorr also has the advantage of more clearly showing the different groups of variables and allows for easy filtering, to view the data with different correlation cutoffs or remove a domain. While filtering by correlation values is a relatively simple task to a user with programming experience, it may be difficult if a user has no such knowledge. Should a user want to examine correlations pertaining to one domain, this is dynamically viewable by hovering over a given domain in GlobeCorr.ca. All manipulations are achievable regardless of the coding experience of the user. GlobeCorr plots scale well for documents and presentations. They are easier to manipulate into a ‘square’ (versus rectangle for many heat maps), allowing for more effective use of white space, and can help users create more engaging and appealing presentations.

In summary, GlobeCorr addresses a widespread need to more dynamically explore complex datasets, including integrated omics datasets, and allows those with limited coding experience to make publication-quality figures, or a dynamic visualization for data exploration.
